# Diagnosis of genital herpes simplex virus infection in the clinical laboratory

**DOI:** 10.1186/1743-422X-11-83

**Published:** 2014-05-12

**Authors:** Jérôme LeGoff, Hélène Péré, Laurent Bélec

**Affiliations:** 1 Université Paris Diderot, Sorbonne Paris Cité, Microbiology laboratory, Inserm U941, Hôpital Saint-Louis, APHP, 1 Avenue Claude Vellefaux, Paris 75010, France; 2 Laboratoire de Microbiologie, hôpital Européen Georges Pompidou, Assistance Publique-Hôpitaux de Paris, Sorbonne Paris Cité, Paris, France; 3 Faculté de Médecine Paris Descartes, Université Paris Descartes (Paris V), Sorbonne Paris Cité, Paris, France

**Keywords:** Herpes simplex virus, Genital herpes, Diagnosis

## Abstract

Since the type of herpes simplex virus (HSV) infection affects prognosis and subsequent counseling, type-specific testing to distinguish HSV-1 from HSV-2 is always recommended. Although PCR has been the diagnostic standard method for HSV infections of the central nervous system, until now viral culture has been the test of choice for HSV genital infection. However, HSV PCR, with its consistently and substantially higher rate of HSV detection, could replace viral culture as the gold standard for the diagnosis of genital herpes in people with active mucocutaneous lesions, regardless of anatomic location or viral type. Alternatively, antigen detection—an immunofluorescence test or enzyme immunoassay from samples from symptomatic patients--could be employed, but HSV type determination is of importance. Type-specific serology based on glycoprotein G should be used for detecting asymptomatic individuals but widespread screening for HSV antibodies is not recommended. In conclusion, rapid and accurate laboratory diagnosis of HSV is now become a necessity, given the difficulty in making the clinical diagnosis of HSV, the growing worldwide prevalence of genital herpes and the availability of effective antiviral therapy.

## Introduction

### Key structure elements for diagnosis

Herpes simplex virus type 1 (HSV-1) and type 2 (HSV-2) are large double-stranded DNA viruses of the Herpetoviridae family, alphaherpetovirinae sub-family [1]. HSV-1 and HSV-2 share a similar genome structure, with 40% of sequence homologies reaching 83% homology of their protein-coding regions, explaining numerous biological similarities and antigenic cross-reactivity between the two types. HSV-1 and HSV-2 genomes each encode at least 80 different structural and non-structural polypeptides including at least 10 different viral glycoproteins of which most are embedded in the viral envelope (gB, gC, gD, gE, gG,gH, gI, gL, gM, gN) [1]. The majority of the antibody response to HSV infection is raised against these surface glycoproteins. Glycoprotein gB, gC, gD and gE trigger potent immune responses. Some epitopes present on these glycoproteins are shared by HSV-1 and HSV-2, and are causing a significant degree of cross reactivity. However, no cross reactivity between glycoprotein gG1 in HSV-1 and gG2 in HSV-2 can be detected [2] which is why antibodies to this glycoprotein are used for type-discriminating serology. While the type common gB and gD display high similarity (85%) the homology betweengG-1 and gG-2 is much lower, presenting an overall amino acids (aa) identity of <30%. The reason for this is that gG-1 of HSV-1 contains 238 aa, while gG-2 of HSV-2 comprises 699 aa [2]. Furthermore, the envelope glycoprotein G (gG-2) of HSV-2 is cleaved into a membrane-bound portion (mgG-2) and a secreted portion (sgG-2). However, the epitopes for the type-specific antibodies against mgG-2 are not located in the portion of mgG-2 which is lacking in gG-1 but in a region with aa similarity to gG-1. This sequence, located between aa 560 and 573 for HSV-2 gG and between aa 80 and 93 for HSV-1 gG, carries nine identical residues between gG-1 and mgG-2 and five type-specific residues that induce significant structural differences. This results in different exposure of key residues utilized for recognition and explains the lack of cross-reactivity [3].

Other similarities and differences between the genomes of HSV-1 and HSV-2 are used also for genera- or type-specific molecular assays, including genes coding for some envelope glycoproteins or the DNA polymerase. The conserved gene coding for DNA polymerase is often used for the detection or quantitation of both types and based on a few mismatches between HSV-1 and HSV-2 sequences which may also be used for typing [4,5].

### Burden of genital herpes

HSV-1 and HSV-2 are ubiquitous, affecting both urban and remote populations worldwide [6]. HSV-1 seroprevalence reaches 50 to 70% in developed countries and 100% in developing countries and HSV-2 seroprevalence varies from 10 to 40% and may reach 60–95% in HIV-infected individuals and female sex workers.

The classical pattern of HSV-1 and HSV-2 infections associated with oral or genital diseases, respectively, remains the rule in certain parts of the world such as sub-Saharan Africa where HSV-1 infection remains a mandatory community acquired disease in childhood, and HSV-2 infection a sexually transmitted infection (STI) in adults. In contrast, the differentiation of HSV-1 from HSV-2 based on anatomical site of infection is far from absolute in developed countries, the proportion of genital ulcers associated with HSV-1 infection has become predominant in some developed countries [6,7]. This is the result of both the delay in acquisition of oral HSV-1 infection early in life in developed countries (rendering a significant proportion of young adults always susceptible to genital HSV-1 infection at initiation of sexual activity) and the oro-genital sexual practices. This feature is concerning in regards to neonatal herpes given that the risk of HSV vertical transmission is higher during primary infection than during reactivation [8] and that HSV-1 appears more readily transmissible to the neonate than HSV-2 [9]. It should be noted that genital HSV-1 infection does not prevent any risks of genital HSV-2 acquisition [9].

Worldwide, HSV-2 remains the main cause of genital herpes and is the major etiology of genital ulcer disease. In addition, HSV-2 infection has been proven to be an independent cofactor of HIV sexual transmission. In turn HIV-1 infection increases the frequency of HSV-2 reactivations and mucosal shedding, as well as the quantity of shed viruses [7]. In severely immunocompromised HIV-1-infected patients and transplant patients, HSV infections frequently present as chronic, necrotic, extended, and confluent mucocutaneous ulcerations.

Most primary genital infections with HSV-1 and HSV-2 are asymptomatic and all are followed by latent infection of neuronal cells in the dorsal root ganglia and only 10–25% of people with HSV-2 antibodies are aware of their genital herpes. However, a large proportion of seropositive patients present asymptomatic shedding episodes that contribute to the spread of these infections [10,11].

### Importance of laboratory diagnosis or testing for genital herpes

**Figure 1:**
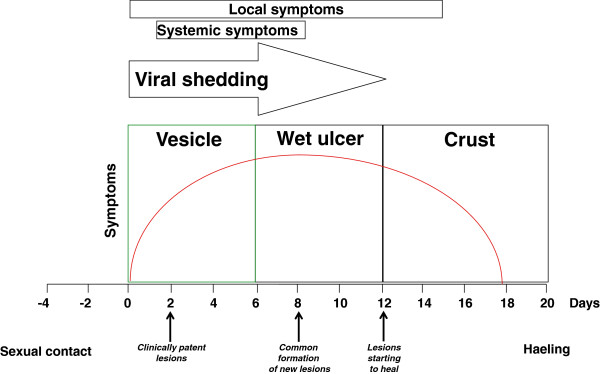
Clinical course of primary genital herpes.

Genital herpetic infection is mainly diagnosed on clinical grounds, especially when the clinical picture is classical, with the presence of typical papular lesions progressing to vesicle and ulcerative lesions which finally crust, associated with local adenitis and in recurrent cases preceded by prodromal [12] (Figure 1). However, clinical diagnosis of genital herpes may be limited in accuracy. The clinical differentiation of genital HSV infection from other infectious (*Treponemapallidum, Haemphilusducreyi*) and non-infectious etiologies of genital ulceration is often difficult and laboratory confirmation of the infection should always be sought [13]. Besides classic vesicular lesions, HSV genital infection may be associated with other clinically atypical presentations. These include either unusual sites (extragenital regions: buttocks, thighs) or atypical morphological forms of genital disease (vulvar, penile or perianal fissures, localized recurrent erythema, recurrent radicular or lower back pain, cystitis, urethritis, vaginal discharge without overt genital lesions) [14,15]. Meningitis may be observed during phases of primary infection and reactivation and can also confuse the diagnosis of genital HSV infection [16]. Accordingly, exclusive reliance on clinical diagnosis could lead both to false positive and false negative diagnosis of the condition. Thus, a clinical diagnosis of genital herpes should be confirmed with laboratory tests [6,12,17-19].

The laboratory diagnosis of genital herpes is recommended in various situations:

• Confirmation of clinically suspected genital herpes.

• Variable presentation of genital herpes.

• Extra-genital complications of genital herpes [20].

• Differential diagnosis with other ulcerative STIs.

• Differential diagnosis with other genital ulcerative dermatoses (Crohn’s disease, Behçet syndrome or fixed drug eruption).

Because HSV-1 has become a frequent etiology of genital herpes, species typing is also a cornerstone of genital herpes diagnosis. Whether genital herpes is caused by HSV-1 or HSV-2 influences prognosis and counseling. Even though up to 50% of first-episode cases of genital herpes are caused by HSV-1, recurrences and subclinical viral shedding are much less frequent for genital HSV-1 infection than genital HSV-2 infection. Thus, information regarding whether one is infected with HSV-1 or HSV-2 can prove useful in discussing risks for recurrence.

### Laboratory methods for direct herpes diagnosis

#### Collection, transport and storage of clinical specimens for herpes diagnosis

HSV-1 and HSV-2 can be recovered by swabbing mucocutaneous genital lesions and from previously involved mucocutaneous sites in patients with asymptomatic infection.

For sample lesions collection, a small cotton, cotton-tipped, or Dacron swab on a wire shaft is used for viral culture as well as molecular biology. Nylon flocked swabs may be preferred since their perpendicular nylon fibers act like a soft brush to allow the improved collection and release from patient samples although no formal validation for herpes positive samples has been performed yet [21-23]. Calcium alginate swabs are toxic to HSV and therefore should not be used for virus isolation in cell culture [24].

For active lesions, collection of vesicular fluid or exudate from small vesicles is the method of choice. After sampling, the specimens for viral culture, antigen or detection of HSV DNA genome should be placed immediately into vials containing 1 ml of appropriate viral transport medium, or an universal transport medium because HSV is highly sensitive to desiccation and pH inactivation. Specimen should also be transferred quickly to a diagnostic virology laboratory on ice (+4°C) as the virus infectivity is heat labile. Molecular assays that do not require the virus infectiousness, tolerate less strict conditions for the sample transport. The use of transport medium may be not necessary as long as samples are stored at +4°C and frozen until molecular analysis. It has been shown that inappropriate storage decreased the yield of HSV DNA [25].The level of viral nucleic acids collected from cervicovaginal lavages remain stable at 4°C for 24 hours but decreased significantly when they were stored at 20°C and 30°C [25].

For a diagnosis using cell culture, the use of alcohol or iodophors to cleanse the lesions before sampling the lesion should be avoided as it inactivates the virus.

The recommended sampling sites and type of sample and methods to be used for the diagnosis of genital herpes infection are presented in Table 1. The recommendations for sample transportation and storage using microscopy, culture and nucleic acid amplifications tests(NAAT) are presented in Table 2.

**Table 1:** **Recommendations for sample collection for the diagnosis of genital herpes infections, adapted from Domeika and colleagues**[9]

**Collection site**	**Tools for sample collection**	**Collection method**
**Male skin or mucous membrane lesions**	• Sterile needles	• Unroof the vesicles with a sterile needle
	• Sterile cotton-tipped, Dacron or nylon flocked swab on a wooden, plastic or aluminium shaft	• Collect the content of the vesicles with a sterile swab and:
		○ apply to a microscope slide (for immunofluorescence staining) or ○ introduce into transport media for viral culture or NAAT.
	• Microscope slides	
**Male urethra**	• Sterile cotton-wool, Dacron or nylon flocked swab on a wooden, plastic or aluminium shaft	• Clean the external urethral opening region with a swab moistened in saline
		• Draw back the prepuce to avoid contamination when sampling
		• Insert a sterile swab carefully into the external urethral meatus (to a depth of 0.5–2 cm) and collect urethral exudates for testing
**Female skin or mucous membrane lesions**	• Gauze and cotton swabs,, dacron or nylon flocked swab on a wooden, plastic or aluminium shaft	• Similarly as for male skin or mucous membrane lesions
	• Microscope slides	
**Female urethra**	• Sterile gauze swab (to remove excess discharge)	• Clean the introitus using a sterile gauze swab
	• Sterile cotton-wool, Dacron or nylon flocked swab on an aluminium shaft	• Carefully insert a sterile swab on an aluminium shaft into the urethra (to a depth of 0.5 cm) to collect exudates for testing
**Cervix**	• Vaginal speculum	• Insert the vaginal speculum, which may be moistened in advance with warm water and
	• Sterile gauze swab	
	• Sterile cotton-wool, Dacron or nylon flocked swab on a wooden or plastic shaft	• clean the cervical canal opening thoroughly with a sterile gauze swab
		• Insert a cotton-wool or Dacron swab carefully into the cervical canal (to a depth of 2 cm) and collect the material from lesions.
**Vagina** **(of prepubertal girls)**	• Sterile cotton-wool, Dacron or nylon flocked swab on an aluminium shaft	• Insert a sterile swab on an aluminium shaft carefully through the hymen into the vagina, and collect the material from the back wall of the vagina
**Urine**	• Sterile container for urine	• Ask the patient to collect the first 10–20 ml of voided urine (first catch)
		• The patients should avoid urinating for least two hours before sampling
**Conjunctiva**	• Sterile cotton-wool, Dacron or nylon flocked swab on an aluminium shaft	• purulent discharge must be removed before sampling with a sterile swab
	• Kimura platinum conjunctival scraper	• Move a swab over the conjunctiva of the inferior eyelid towards the interior angle of the eye (use a thin swab on an aluminium shaft for newborns)
	• Topical ophthalmic local anaesthetic	
		• The Kimura scraper is used to sample the bases of lesions (either ulcers or the bases of burst vesicles). Before collecting the sample, the spatula is sterilised by heating in a flame and allowed to cool
**Rectum**^**a**^	• Rectal speculum or proctoscope	• Rectal material is taken under direct vision, with the aid of a proctoscope or rectal speculum. Use of a blind technique results in considerable loss of sensitivity
	• Sterile cotton-wool, Dacron or nylon flocked swab on a wooden or aluminium shaft	
		• Insert a swab on a wooden or plastic shaft to a depth of 3 cm and collect the material from all rectal walls by circular motions for 10 seconds
		• If faecal material is impacted, the swab should be discarded and the sampling procedure repeated.

^a^Material from the rectum is collected when the patient has had anal sexual contact, when he suffers from anorectal inflammation, or if perianal skin or anal folds are thickened.

NAAT: nucleic acid amplification test.

**Table 2:** **Recommendations for sample transportation accordin g to the test method, adapted from Domeika and colleagues**[9]

**Test method**	**Conditions**	**Comments**
**Viral culture**	• Immediately after sampling the material must be placed in appropriate transport medium, such as Eagle’s medium with addition of antibiotics	• Herpes simplex virus is sensitive to both the temperature and to drying out
	• The material should preferably be transported to the laboratory on ice, and kept at °4°C for up to 48 hours	
	• Material should not be kept for more than 4 hours at room temperature	
	• Accurately marked test tubes must be placed in a hermetic reservoir and transported to the laboratory accompanied by the relevant documentation including the investigation method requested	
**Antigen detection and nucleic acid amplification tests**	Transport medium is usually provided by the manufacturer of the diagnostic commercial assay	• The material is generallly delivered in special test tubes with transport medium according to the manufacturer’s instructions for each test
	• If the sample transportation procedure is not described in the manufacturer’s instructions or in-house test systems are used, transportation is performed as follows:	
	oClinical material placed in univesal transport medium should be transported in a cool bag at 4 ± 2°C	
	oUrine should be delivered to the laboratory within three hours of collection, at ambient temperature	
	• Test tubes containing clinical material should be transported to the laboratory accompanied by the relevant documentation including the investigation method requested	
**Microscopy (direct examination or immunofluorescence)**	• If there is a need to save the material for more than 24 hours, the smear should be fixed with 96% ethyl alcohol for three minutes	• If the rules of sampling and conditions of transportation of the biological material are not followed (*e.g.* slides are broken, unmarked or stuck together or there is no material on the slide), microscopy examination should not be carried out
	• Each smear on a microscope slide should be placed in the transportation container and transported to the laboratory accompanied by the relevant documentation including the investigation method requested	
		• Method rarely used now

NAAT: nucleic acid amplification test.

#### Laboratory methods for direct herpes diagnosis

Several tests with various specificities and sensitivities are used for the direct diagnosis of HSV infections (Table 3).

**Table 3:** Direct laboratory methods for HSV diagnosis

**Method**	**Principle**	**Sample**	**Sensitivity**	**Specificity**	**Advantages**	**Disadvantages**
**Viral antigen detection**	Immunopreoxidase staining	Swab	Middle (80%)	High (90%)	Reagent cost	Fresh vesicles
		Smears from lesions				
					Rapid (<4 hours possible)	Suboptimal sensitivity
		Smear or vesicular fluid of exudate from base of vesicle				
					Does no require the integrity of the specimen	
					Typing possible	
	Capture ELISA	Swab	High (Genital ulcer: >95%)	High (62-100%)		Fresh vesicles
		Vesicular fluid or exudate from base of vesicle				No viral typing
	Rapid test device	Swab	Unknown	Unknown	Point-of-care testing	Not yet evaluated
		Vesicular fluid or exudate from base of vesicle				
**Virus culture**	HSV isolation susceptible cells	Swab	Low to high depending of the clinical context	High (≈100%)	Allows virus isolation	Less sensitive than PCR
		Skin lesions				
						Sample storage and transport conditions influence sensitivity
					Classically, “gold standard” method	
		Vesicular fluid or exudate from base of vesicle	Vesicular content :>90%			
						(**➜** Rapid transport, cooled, protected from light in virus transport medium)
					Currently, “preferred” test (CDC 2010)	
			Ulcer : 95%			
			Swab : 70%-80%			
						Labor-intensive
		Mucosal sample without lesions Biopsies	Mucosa without lesion: 30%		Simplicity of sampling	
						Expensive
					Virus typing	Specialized laboratories
					Resistance	Results in 2/7 days
					Phenotype testing*	Arrangement with laboratory necessary
		Conjunctival/corneal smear				
		Neonates				
**Molecular biology**	HSV DNA detection and/or quantitation by NAAT, including in-house classical PCR, real-time PCR and commercial assays	Swab	Highest	High.	High sensitivity.	Only in specialized laboratories
		Skin lesions	(98%)	(≈100%)		
		Vesicular fluid or exudate from base of vesicle		Containment of potential cross-contamination important	Currently, “preferred” test (CDC 2010)	Not standardized
					Allows virus detection and typing in the same test	Not validated for all samples
		Mucosal sample without lesions				
						Risk of contamination (PCR)
						May be relatively expensive (real-time PCR)
					Rapid	
		Aqueous/vitreous humor			May be automated.	
					Labor efficient	Routine resistance genotyping not available
		Cortico-spinal fluid				
					Result within 24–48 h, possibly in <3 hours	
		Blood				
					Resistance genotyping	
					Method of choice for CSF	
					**Real-time PCR:**	
					Rapid amplification	
					Quantitative analysis	
					Reduced risk of contamination	
					Method of choice for skin lesions	
**Cytological examination**	Tzanck smears	Skin/mucosal lesions	Low	Low	Inexpensive	Fresh lesions
	Papanicolaou or Romanovsky stain					low sensitivity and no distinction between HSV-1 and HSV-2, nor between HSV and varicella zoster virus infection
		Biopsies				
		Conjunctival/corneal smears				
	Detection of infected cells by direct immunoflorescence	Smears, Tissue section Smear from base of vesicle	Middle	High	Inexpensive	Fresh vesicles
			(Genital ulcer: 70-90%	(>95%)	Rapid (<4 hours possible)	Suboptimal sensitivity
			Asymptomatic : < 40-50%)			
					Typing possible	Time-consuming
						Labor-intensive
						Not standardized

*The detection of resistance mutations to anti-herpetic drugs (aciclovir) by HSV drug resistance genotyping is likely to supplant phenotypic testing in the next few years.

NAAT: nucleic acid amplification test; CDC : Centers for Disease Control.

Viral culture with further herpes typing has been the cornerstone of HSV diagnosis over the past two decades and accepted as the gold standard for the laboratory diagnosis of HSV infections.

Viral antigen can be easily detected by direct immunofluorescence (IF) assay using fluorescein-labelled type-specific monoclonal antibodies on smears, or by enzyme immunoassay (EIA) on swabs. Although these assays lack sensitivity, they perform satisfactorily in symptomatic patients. Thus, these direct methods may offer a rapid diagnostic alternative in settings where laboratory facilities are limited, including resources-constrained countries.

Recently, HSV DNA detection based on nucleic acid amplification, and polymerase chain reaction (PCR) in particular, has emerged as an alternative method because it is about four times more sensitive, less dependent on collection and transport conditions, and faster than viral culture [26]. The 2010 CDC Sexually Transmitted Diseases Treatment Guidelines state that “PCR testing to diagnose herpes can be performed by clinical laboratories that have developed their own tests and have conducted a Clinical Laboratory Improvement Amendment (CLIA) verification study”, and “cell culture and PCR are the preferred HSV tests for people who seek medical treatment for genital ulcers or other mucocutaneous lesions” (CDC, 2010). Since 2011 three molecular assays have been approved by the US Food and Drug Administration for the testing of genital specimens (IsoAmp HSV Assay, BioHelix Corporation; MultiCode-RTx Herpes Simplex Virus 1 & 2 Kit, EraGen Biosciences, Inc.; BD ProbeTec Herpes Simplex Viruses (HSV I & 2) QX Amplified DNA Assays, BD Diagnostic Systems).

Based on our practice, when molecular testing is available, its use should be preferred over viral culture. Molecular testing will also confirm viral shedding whether or not lesions are present [27]. When no facilities are available to carry out cell culture or molecular assays, antigen detection is useful and can provide a rapid diagnosis, mainly when mucocutaneous lesions are present.

The recommended sites and methods to be used in the direct diagnosis of genital herpes lesions are presented in Table 4.

**Table 4:** **Recommended sampling sites, type of sample and preferred diagnostic methods for genital herpes, adapted from Domeika and colleagues**[9]

**Sampling site or type of sample**	**Preferred diagnostic method**
Vesicule on skin and mucous membranes Ulcer	NAAT; viral culture; antigen detection*
Urethra (male)	NAAT; antigen detection*
Cervix/urethra (female)	NAAT; antigen detection*
Urine (men and women)	NAAT; viral culture
Vulva/vagina (prepubertal girls)	NAAT
Vagina (women after hysterectomy)	

*Viral antigen detection by direct immunofluorescence on smears or enzyme immunoassay on swabs may offer a rapid diagnostic alternative in settings where culture or molecular diagnosis are not available.

NAAT: nucleic acid amplification test.

#### Virus isolation and typing in cell culture

Several primary, diploid and continuous cell lines may be used for isolation of HSV from clinical specimens. Commonly used cells, sensitive to different viruses, include mainly primary human diploid fibroblasts, such as MRC-5 cells, and cell lines, such as Vero cells (monkey kidney), HEp-2 cells (laryngeal squamous cell carcinoma), baby hamster kidney and rabbit kidney cells [28]. The parallel inoculation of two different cell lines can minimize the effects of periodic variations in cell line sensitivity.

Culture cells are first allowed to grow into a confluent monolayer in a tissue culture tube flattened on one side. The cytopathic effect (CPE) caused by HSVusually develops 24-72 hours after inoculation, and is characterized by enlarged, refractile, and rounded cells. Focal necrosis of cells may occur and syncytia and multinucleated giant cells may be present. Within days, the monolayer may be destroyed. The incubation time required to observe the cytopathic effect of HSV depends on the concentration of the virus in the clinical specimen: samples with high titers of virus produce CPE in less than 48 hours, whereas samples with a low concentration produce CPE after 4-6 days. Cultures should be held for seven to 10 days. The highest isolation rates of HSV are likely if the clinical specimens are inoculated on the day they are taken. It is important to give attention to the conditions of transport and storage of clinical specimens. They must be stored at +4°C during transport and maintained at this temperature for no longer than 48 hours. At ambient temperature, transport duration should be less than 4 hours. If a delay of more than 48 hours is expected between collection and culture, the specimens should be frozen at best at -80°C for further inoculation. Virus titers are remarkably reduced in frozen and thawed samples, and freezing at -20°C is not advised [25].

Confirmation of HSV in viral culture demonstrating cytopathic effect is recommended since other viruses may exhibit a cytopathic effect similar to that observed in herpes culture, and allows viral typing. Typing of HSV using cell culture can be performed directly on infected cell cultures using fluorescein-labelled type-specific monoclonal antibodies by direct immunofluorescence which constitutes the most practicable procedure, or, eventually, by testing the cell supernatant by molecular assays [28].

As standard virus isolation in tissue culture may be slow, in particular for samples with low viral titers, many laboratories now use centrifugation-enhanced (shell vial) culture methods combined with staining with a type-specific monoclonal antibody before the CPE onset to reduce viral isolation times [29,30]. Shell vial culture can reduce viral isolation time from one to seven days to just 16-48 hours.

Genetically engineered cell lines have been developed to allow an early detection of HSV-1 and HSV-2 after an overnight incubation. The Enzyme Linked Virus Inducible System (ELVIS, Diagnostic Hybrids, Inc, USA)utilizes genetically engineered cell lines transfected with an inducible HSV promoter gene linked to an*Escherichia coliLacZ* reporter gene [31]. Replication of HSV in these cells induces galactosidase production, and infected cells stain blue when overlaid with an appropriate substrate [32]. Typing can then be performed using type-specific antisera on any monolayers showing blue cells.

Diagnosis of HSV infection with tissue culture has low sensitivity because HSV is isolated from lesions in about 80% of primary infections but in only 25–50% of recurrent lesions, and in even fewer people whose lesions have begun to heal. Thus, fluid collected from intact blisters (vesicular or pustular lesions) will grow out in culture more than 90% of the time. By the time the lesions have crusted over, only about 25% of cultures will be positive. Failure to detect HSV by culture does not indicate an absence of HSV infection [26].

#### Antigen detection

Viral antigen can be easily detected by direct or indirect immunofluorescence (IF) assay using fluorescein-labelled type-specific monoclonal antibodies on smears, or by enzyme immunoassay (EIA) on swabs. For detecting HSV in lesions, the sensitivity of antigen detection tests may be the same as that of culture assay but is lower than nucleic acid amplification test sensibility [4]. As indirect IF assay and EIA perform satisfactorily in symptomatic patients, these direct methods may offer a rapid diagnostic alternative in settings where laboratory facilities are limited and where specimen handling and transportation conditions could inactivate the virus. This is true for remote locations where prolonged specimen transport time under inappropriate conditions may occur before delivery to the microbiology laboratory.

For immunofluorescent assays, the slide should be prepared by the laboratory using a cytospin method to guarantee the quality of the slide reading. Under a fluorescence microscope, infected cells will be recognized by the presence of a characteristic pattern of apple-green fluorescence in the nucleus and cytoplasm of the basal and parabasal cells.

Several EIA assays are commercially available but few have been FDA approved.

#### Virus detection and quantification by molecular biology

Molecular biology has emerged for the last ten years as an attractive potent method to detect and possibly quantify HSV DNA. Most of NAATs are based on the PCR but some use a different approach for the amplification of nucleic acid.

Several procedures have been proposed to detect and/or quantify HSV genomes in clinical samples, including in-house competitive PCR [33], PCR detection followed by DNA enzyme immunoassay hybridization [34], real-time PCR assay [4,5,35,36], and various commercially available kits. The majority of in-house or commercial PCR targeting the HSV genome are currently based on real-time PCR which allows both the detection and the quantification of HSV DNA in clinical samples. Compared with traditional PCR (also called end-point PCR) revealed either with agarose gel migration or enzyme hybridization assay, real-time PCR is faster, less labor-intensive with minimal technical hands-on time and a lower risk of molecular contamination. Primers from HSV DNA sequence common to both HSV-1 and HSV-2 [HSV DNA polymerase, HSV thymidine kinase or glycoprotein B] may identify HSV DNA. In some assays, a melting curve at the end of real-time PCR helps discern HSV-1 from HSV-2 [4,5,36]. Primers and probes from HSV DNA sequence specific to HSV-1 or HSV-2, including, gB, gD, or gG genes, allows also the amplification of one specific herpes type [35,37-40]. In each experiment positive and negative controls should be run. In addition, the use of internal controls spiked before nucleic acid extraction is recommended to detect the presence of any amplification inhibitors that could lead to false-negative results.

PCR assays or other NAATs are the most sensitive test currently available to detect HSV in clinical samples. The detection rates of the PCR assays were shown to be 11–71% superior to virus culture [26,41-44]. Furthermore, NAAT allows the best detection of asymptomatic shedding of genital herpes beside symptomatic infections [26]. However, failure to detect HSV by PCR does not indicate an absence of HSV infection, because viral shedding is intermittent [11].

Three NAATs have been approved by the US Food and Drug Administration for the testing of genital specimens (IsoAmp HSV Assay, BioHelix Corporation; MultiCode-RTx Herpes Simplex Virus 1 & 2 Kit, EraGen Biosciences, Inc.; BD ProbeTec Herpes Simplex Viruses (HSV I & 2) QX Amplified DNA Assays, BD Diagnostic Systems).

The IsoAmp HSV Assay uses isothermal helicase-dependent amplification in combination with a disposable, hermetically-sealed, vertical-flow strip identification, limiting the technical hands-on time and risk of cross-contamination. Once DNA is purified from the sample, the assay has a total test-to-result time of about 1.5 hours. The diagnostic sensitivity and specificity are comparable to end-point PCR and are superior to culture-based methods. The performances have not been compared to real-time PCR assays. The assay is FDA approved for the detection of herpes simplex viruses (HSV) in genital and oral lesion specimens. The assay does not provide specific typing information to differentiate HSV-1 from HSV-2. The assay is not intended to be used for prenatal screening [45].

The MultiCode-RTx Herpes Simplex Virus 1 & 2 Kit utilizes real-time PCR molecular detection. MultiCode-RTx technology site-specifically incorporates an isoG triphosphate, covalently attached to a DABCYL quencher, opposite an isoC base that is adjacent to a 5′ fluorescent label in one of the primers. PCR amplification is performed using the Roche LightCycler 1.2 instrument. Incorporation of the quencher-labeled nucleotide causes a decrease in assay fluorescence when the product is a double-stranded DNA molecule. The PCR primers target a type-specific DNA sequence within the herpes simplex virus glycoprotein B gene. The MultiCode-RTx Herpes Simplex Virus 1 & 2 Kit is indicated for use in the detection and typing of HSV-1 or HSV-2 in vaginal lesion swab specimens from symptomatic female patients. The assay provided similar sensitivity and specificity compare to two other commercial real-time PCR assays on CSF samples [46].

The BD ProbeTec Herpes Simplex Viruses (HSV-1 & -2) QX Amplified DNA Assay is a fully automated assay for HSV-1 and HSV-2 molecular detection and typing on the BD Viper™ System. The PCR primers target a type-specific DNA sequence within the HSV glycoprotein G gene. It is approved for the detection and differentiation of HSV-1 and HSV-2 in anogenital samples. It has been compared to HSV culture and a laboratory-developed real-time PCR assay with 508 clinical specimens. The sensitivity of HSV-2 detection ranged from 98.4-100% depending on the analytical approach, while the specificity ranged from 80.6%, compared to the less sensitive culture method, to 97.0%, compared to PCR. For HSV-1, the sensitivity and specificity ranges were 96.7-100% and 95.1-99.4%, respectively [47].

### Indirect serological diagnosis of herpetic infections

Detection of HSV-specific IgG antibodies can be done sensitively by several immunological methods. Serologic diagnosis of HSV infections and HSV type-specific antibody testing are summarized in Table 5, and commercially available assays approved by the Food and Drug Administration (FDA, United States) in Table 6. Accurate type-specific HSV serologic assays are based on the detection of HSV-specific gG1 (HSV-1) and gG2 (HSV-2) antibodies using native, purified or recombinant gG1 or gG2 as antigens. Serological assays based on antigen preparations from whole virus or from crude infected-cell protein mixtures detect predominantly type-common antibodies, may have low sensitivity in detecting HSV-2 antibodies in HSV-1–seropositive patients, or may incorrectly type antibodies in patients with only HSV-1 or HSV-2 infection. Some commercial assays described as “type-specific” are actually based on relative reactivity of serum antibodies to crude preparations of HSV-1 versus HSV-2 antigens. The accuracy of such tests for HSV-2 antibody detection is low compared with glycoprotein G–based tests, and their use is not recommended [48].

**Table 5:** Indirect serological assays for HSV diagnosis

**Method**	**Principle**	**Sample**	**Sensitivity**	**Specificity**	**Advantages**	**Disadvantages**
**Western blot**	Western blot HSV-1	Serum	≈100%	≈100%	Reference (“gold standard”) test proposed by University of Washington (USA)	Not commercially available
						Expensive
					[UW-WB]	
					Specific of HSV-1 and HSV-2	2–3 days for results
	Western blot HSV-2					
					Detect early sero-conversion to HSV-2 in patient with prior HSV-1 infection	
					Earliest sero-conversion : 13 days	
**Enzyme immune-assay**	Monoclonal antibody-blocking EIA	Serum’	≈100%	≈100%	Reference (“gold standard”) test proposed by the Central Public Health Laboratory in the United Kingdom; 98% concordance with WU-WB	Not commercially available
			(African sera : 98%)	(African sera : 97%)		
					Distinguish between HSV-1 and HSV-2	
**Enzyme immune-assay**	ELISA	Serum	93–98%	93–99%	Commercially available	May lack of sensitivity and specificity
					Distinguish between HSV-1 and HSV-2	Lack of specific on African sera
**Point of care tests**	Immuno-filtration	Serum Capillaryblood	96%	87–98%	Less expensive than Western blot	Commercially available only for HSV-2
					Accurate results rapidly (6 min.)	Expensive
						Not for large volume screening
					Easily to carry out	
					Detects seroconversion within 4 weeks of presentation of 80% of patients with HSV-2 episodes	Complexity nonwaived (moderate)

ELISA:Enzyme-linked immunosorbent assay; EIA: Enzyme immunoassay;

UW-WB: Western blot test developed at the University of Washington.

**Table 6:** Commercially available serological assays for HSV diagnosis approved by the Food and Drug Administration (US) (FDA, 2013)

				**HSV-1**		**HSV-2**	
**Assay**	**Manufacturer**	**Format**	**Collection method**	**Sensitivity**	**Specificity**	**Sensitivity**	**Specificity**
**Biokit HSV-2 Rapid Test**	Biokit	Point of care	Heparinized capillary whole blood, serum	NA	NA	93%-96%	95%-98%
**HerpeSelect HSV-1 and HSV-2 Immunoblot**	Focus Diagnostics	Western blot with recombinant proteins	Serum	99.3%	95.1%	97.3%	93.7%
**HerpeSelect HSV-1 ELISA, HerpeSelect HSV-2 ELISA**	Focus Diagnostics	ELISA	Serum	91.2%-96%	92. 3%-95.2%	96.1% -100%	97.0%-96.1%
**CaptiaHsv 1 IgG Type Specific Elisa Kit&CaptiaHsv 2 IgG Type Specific Elisa Kit**	Trinity Biotech	ELISA	Serum	87.9%-87.7%	100%-98.2%	96.7%-100%	90.3%-91.5%
**Liaison HSV-1 & Liaison HSV-2 Type SpecificIgGAssay**	Diasorin	ELISA	Serum	96.9%-98.7%	91.3%-96.8%	98.1%-94.8%	98.0%-97.3%
**Zeus ELISA HSV GG-2 IgG Test System & Zeus ELISA HSV GG-1 IgG Test System**	Zeus Scientific	ELISA	Serum	96.8%	97.1%	98.8%	100%
**BioPlex HSV-1 & HSV-2 IgG panel**	Biorad	Luminex	Serum, lithium heparini plasma, EDTA plasma	100%-100%	98. 3%-97.4%	99.4%-100%	100%-100%
**Elecsys HSV-1 IgG and HSV-2 IgG assays**	Roche Diagnostics	Chemiluminescence	Serum, lithium heparin plasma, EDTA plasma	94.2%-91.0%	90. 3%-95.7%	93.6%-97.8%	98.7%-98.7%

NA: Not Applicable.

Type-specific IgG antibodies are negative in early presentations of herpes disease, and become detectable two weeks to three months after the onset of symptoms and persist indefinitely. Thus, immediately after infection there is a ‘window’ in which testing for antibodies will give a negative result. Consequently, primary HSV infections can be documented by using any serologic methods to show seroconversion with paired sera. HSV IgM testing substantially increased the ability to detect early infection in patients who lack detectable IgG, but may be negative during primary disease. IgM testing can also be positive during reactivation of disease and cannot be used to distinguish primary from recurrent infection. Because of these limitations, HSV IgM testing has limited availability in routine diagnostic settings and cannot be recommended in routine clinical practice.

Gold standard noncommercial tests for HSV-2 include the immunodot enzyme assay (developed at Emory University, Atlanta, Georgia, United States), the Western blot test (developed at the University of Washington (UW-WB)), and the monoclonal antibody-blocking enzyme immunoassay (developed by the Central Public Health Laboratory, London, United Kingdom) [49,50]. These tests are used in their respective specialized reference laboratorie**s** but are not replicable in many settings, thereby limiting their suitability for large-scale epidemiologic studies. The UW-WB test has been used as a gold standard in several studies, including the evaluation of commercial serological assays required for clearance by the FDA, and in the evaluation of the performance of other gold standard tests. Despite its excellent performance, Western blot remains primarily a research tool. At present, Western blot is not FDA approved, and requires a high level of technical ability, time, and expense to perform.

Type-specific HSV glycoprotein G (gG)-based ELISA became commercially available in 1999. The sensitivities of these gG type-specific tests for the detection of HSV-2 antibody vary from 80–98%, and false-negative results might be more frequent at early stages of infection [51]. The specificities of these assays are ≥96%. The tests approved for use in the USA have sensitivity of 97–100% and specificity of 94–98%, when measured in comparison with the Western blot. False-positive results can occur, especially in patients with a low likelihood of HSV infection. Repeat or confirmatory testing might be indicated in some settings, especially if recent acquisition of genital herpes is suspected [51]. Some HSV-2 strains have been identified with mutations or deletions in gG2-gene leading either to the lack of gG-2 expression or the production of truncated forms [52,53]. Infections with such variants caused genital lesions similar to wild HSV-2 infection but immune response to gG-2 were either reduced or absent [52,53]. Negative detection of type-specific HSV-2 antibodies does not eliminate the rare possibility of a HSV-2 infection. HSV-2 DNA detection or HSV-2 isolation in cell culture along with a negative serology beyond the primary infection suggests an infection with a gG-2 deficient HSV-2 strain.

HerpeSelect® ELISA tests (HerpeSelect® 1 ELISA IgG Herpes Simplex Virus-1 (HSV-1) ELISA IgG; HerpeSelect® 2 ELISA IgG Herpes Simplex Virus-2 (HSV-2) ELISA IgG,Focus Technologies, Inc., Cypress, CA [formerly MRL Diagnostics]) are FDA approved, widely available and have been extensively studied [51,54-57].

Point-of-care rapid tests can also provide results for HSV-2 antibodies from capillary blood or serum during a clinic visit. These immunoassays are designed to use capillary blood from a finger stick (or serum) and typically employ lateral flow of serum through a membrane containing a dot of gG1 or gG2 antigen. When serum is applied to the kit, a visual color change develops (pink dot) if herpes antibodies are present. Despite a reported inter-operative variability of 5-10% in test interpretation, these point-of-care tests perform relatively well with sensitivities ≥91% and specificities ≥94% [48]. The major benefit of point-of-care assays is that they give results rapidly (potentially while the patient is still in the clinical site,) allowing for more timely patient education and counseling. The major drawback of these tests is their cost relative to herpes ELISA-based systems.

If genital lesions are present, type-specific serology and direct virus testing can help to establish if the episode is a new HSV infection or reactivation (Table 7).

**Table 7:** **Virological and serological approach to HSV-2 diagnosis in the presence and absence of genital lesions, adapted from Gupta and colleagues**[5]

	**HSV-2 detection by direct method**	**HSV-1-specific IgG**	**HSV-2-specific IgG**	**Interpretation**
**First assessment of genital lesions**	Positive	Positive or negative	Negative	Acute HSV-2 infection
				Repeat HSV-2-specific serology within 15-30 days
	Positive	Positive or negative	Positive	Recurrent HSV-2 infection with HSV-2 infection acquired at least 6 weeks ago
**No lesions**	NA	Negative	Negative	Patients at risk for acquiring orolabial or genital HSV-1 infection and/or HSV-2 infections
	NA	Positive	Negative	Patients at risk for acquiring orolabial or genital HSV-2 infections
	NA	Positive	Positive	HSV-1 and HSV-2 past-infections
**Recurrentgenitallesions**	Positive	Positive or negative	Positive	Recurrent HSV-2 infection
	Negative	Negative	Positive	Possible recurrent HSV-2 infection Other potential causes of genital ulcerative disease should be considered

NA: Not applicable.

Type-specific HSV antibodies can take from 2 weeks to 3 months to develop. Thus, in a person with newly acquired herpes the initial absence of IgG antibodies specific for gG and subsequent development of such antibodies after 12 weeks confirms new HSV infection. The distinction between newly acquired HSV and reactivated HSV is helpful for epidemiological studies, and is sometimes helpful clinically for management of psychosocial issues, because it can help clarify the source of infection.

Because nearly all HSV-2 infections are sexually acquired, the presence of type-specific HSV-2 antibody implies anogenital infection; thus, education and counseling appropriate for people with genital herpes should be provided. The presence of HSV-1 antibody alone is more difficult to interpret. The majority of people with HSV-1 antibody have oral HSV infection acquired during childhood, which might be asymptomatic. However, acquisition of genital HSV-1 appears to be increasing, and genital HSV-1 also might be asymptomatic.

Taken together, type-specific HSV serological assays might be useful in the following situations (Table 8):

• Recurrent genital symptoms or atypical symptoms with negative HSV cultures;

• Clinical diagnosis of genital herpes without laboratory confirmation;

• Partner with genital herpes.

**Table 8:** Indications of type specific serology

**Context**	**Indication and interpretation**
Asymptomatic patients	Not routinely recommended
Confirmation of clinical diagnosis	HSV-2 antibodies are supportive of a diagnosis of genital herpes.
History of recurrent or atypical genital disease with direct virus detection negative	HSV-1 antibodies do not differentiate between genital and oropharyngeal infection.
	Counseling of HSV-2 IgG-negative, HSV-1 IgG-positive patients should take into account that HSV-1 is an uncommon cause of recurrent genital disease.
First-episode genital herpes	Differentiation between primary and established infection guides counseling and management.
	At the onset of symptoms, the absence of HSV IgG against the virus type detected in the genital lesion is consistent with a primary infection.
	Seroconversion should be demonstrated at follow-up.
Partner with genital herpes	Knowledge of infection status can guide patient education and counseling if the partnership is discordant.
Pregnant women	Not routinely recommended.
	HSV-1 and/or HSV-2 seronegative women should be counseled about strategies to prevent a new infection with either virus type during pregnancy.
HIV infected patients	Not routinely recommended.
	Although HSV-2 seropositivity increases the risk of HIV transmission and frequent HSV recurrences augment HIV replication, there is limited evidence to inform the management of HSV-2 co-infection in HIV-infected patients without symptoms of genital herpes.
	Limited data suggest an increased risk of perinatal HIV transmission among HSV-2 seropositive HIV-infected women. As the evidence is not consistent, testing of HIV-positive pregnant women is not routinely recommended.

In addition, HSV serologic testing should be included in a comprehensive evaluation for STIs among people with multiple sex partners, HIV infection, and men who have sex with men who are at increased risk for HIV acquisition. Screening for HSV-1 or HSV-2 in the general population is not recommended, due to concerns that HSV-2 diagnosis provides no benefit and could lead to psychosocial sequelae (Table 8).

However some data suggest that most people are interested in HSV-2 testing, which may result in safer sex practice. A review examined studies that measured the short and long-term psychosocial effects resulting from serological diagnosis of HSV-2 in persons without recognized symptoms of genital herpes infection [58]. Overall HSV-2 serological testing did not result in long-term psychosocial harm in most people. Recently a study conducted in pregnant women showed that serotesting sexual partners of pregnant women for HSV reduced the frequency of unprotected genital sex acts in pregnant women at known risk of HSV-2 acquisition compare to HSV-2-seronegative women with partners who were negative or not tested [59].

### Therapeutic monitoring: drug resistance testing

Long-term prophylaxis and treatment with antiviral drugs targeting the viral DNA polymerase (DNA pol) can result in the development of resistance [60]. The prevalence of acyclovir-resistant HSV is about 1% in immunocompetent individuals and increases in immuncompromised patients, 5% in HIV-seropositive individuals and 30% in hematopoietic stem cell recipients [61,62]. Antiviral drugs such as acyclovir or valacyclovir inhibit the viral DNA pol in triphosphorylated form, the first phosphorylation step ensured by the viral thymidine kinase (TK) and the subsequent steps by host cell kinases. Therefore mutations in both DNA pol and TK may confer resistance to antiviral drugs (Table 9). Because a functional TK may be dispensable but not the DNA pol for HSV replication, there is a higher probability of inducing a viable acyclovir-resistant virus by a mutation in the UL23 gene coding for TK than by a mutation in the UL30 gene coding for DNA pol. Accordingly, 95% of clinical isolates exhibiting acyclovir resistance harbor mutations in UL23 gene [61,62].

**Table 9:** **Molecular changes associated with anti-herpetic drugs resistance in thymidine kinase (TK) and DNA polymerase (DNA pol) genes of Herpes simplex virus type 1 (HSV-1) and type 2 (HSV-2) according to amino acid mutations, stop codon and nucleotide insertion or deletion reported in the literature**[29-34]

**Gene**	**Drug**	**Aminoacidmutations**^**a**^	**Stop codon**^**a**^	**Nucleotide insertion/deletion**^**b**^	**Association of mutations**^**a**^
**HSV-1 TK**	**ACV**	R51W, Y53P/D/H, D55N, G56S/V, P57H, K62N, H58R/L, G59R/Y/W, G61V, K62N, T63A/I/S, T64A/S, T65N, E83K, P84S, V87H, T103P, Q104H, H105P, Q125E/L, M128A/F, G129D, G144N/R, A156V, D162A, R163H/C, A167V, A168T, L170P, Y172C/F, P173L/R, A174P, A175V, R176Q, L178R, S181N, Q185R, V187M, A189V, G200C/D, T201P, G206R, L208H, R216C/H/S, R220C/H, R222C/H, L227F, Y239S, T245M/P, T287M, L297S, L315S, C336Y, L364P	Y53, S74, E95, T103, Q104, R176, Q250, Q261, R281, L341, C336, Q342, L364, A375	133-136, 153-155, 180-183, 184-187 430-436, 437-438,455-458, 460-464, 464-465, 548-553,615-619, 666-669, 853-856, 878-880, 896-900, 1061-1064	
**HSV-2 TK**	**ACV**	R34C, R51W, G56E, G59P, P85S, N100H, Q105P, T131P, R177W, S182D, S182N, V192M, T202A, R217H, R221H, R221C, R223H, L228I,D229H, R272V, P273S, D274R, T288M,C337Y	A28, L69, D137, Q222, Y240, T264	215-217, 219-222222, 439-440, 452, 467, 519-521, 551-556, 586-591, 626-628, 808-812	R272V + P273S + D74R
					P85S + N100H + V192M
**HSV-1 DNA pol**	**ACV**	D368A, E370A, V462A, K532T, Y557S, Q570R, D581A, G597K/D, A605V, Q618H, Y696H, R700G, L702H, V714M, V715M, F716L, A719V/T, S724N,E771Q, L774F, L778M, D780N, L782I, P797T, E798K, L802F, V183M, N815L/S/T/V/Y/E, Y818C, T821M, G841S/C, R842S, S889A, F891C/Y, V892S, D907V, I922N/T, Y941H, V958L, R959H, N961K, D1070N			A719V + V904M
					A327T + A605V
					T566A + A605V
	**FCV**	N494S, A605V, F716L, A719V,A719T, S724N, L778M, D780N, L782I, E798K, F891C, D907V, V958L			A719V + V904M
					A327T + A605V
					S724N + A916V
	**CDV**	A136T, R700H, R700M, S724N, T821M, L1007H, I1028T			
	**ACV + FCV**	A605V, F716L, A719V, A719T, S724N, L778M, D780N, L782I, E798K, F891C, D907V, V958L			A719V + V904M
					A327T + A605V
	**ACV + CDV**	T821M			
**HSV-2 DNA pol**	**ACV**	E250Q, R628C, E678G, A724V, S725G, D785N, D912N/V			
	**FCV**	S725G, S729N, L783M, D912V			
	**ACV + FCV**	S725G, D912V			

^**a**^The number is the amino acid position in the protein. The two letters correspond respectively to the wild type amino acid and the mutated amino acid.

^**b**^Nucleotide numbering TK: thymidine kinase, DNA pol: DNA polymerase, ACV: aciclovir, FCV: foscarnet, CDV: cidofovir.

Resistance may be suspected when lesions persist for more than 1 week after initiating antiviral treatment or the emergence of new satellite lesions during treatment. A virological confirmation helps health care professionals choose among different treatment options while it avoids the selection of multidrug-resistant strains [63].

Resistance can be assessed by the detection of specific mutations in UL23 or UL30 genes conferring resistance to antiviral drugs (genotypic assays) or by testing a virus against antiviral agents (phenotypic assays). Because most resistance cases are due to TK deficiency or to defective TK activity, mutations in the UL23 gene should be tested first. Genotypic assays consist of the comparison of UL23 and UL30 genes sequences with the whole panel of mutations described in the literature (Table 9) [61-67]. To be useful in clinical practice, it is essential to be able to discriminate between random variations (polymorphism) and true drug resistance mutations. Therefore, when possible, it is best to test in parallel strains collected before and on antiviral therapy. Before starting genotypic assays, an estimation of the viral load should be obtained because the amplification may be hampered at low levels, especially for the UL30 gene that has been shown to need more than 4.5 and 5.5 log10 copies/ml for HSV-1 and HSV-2 respectively. Virus isolation in cell culture may be required to increase the input of DNA material [64]. However amplification in cell culture can alter the population balance in the native sample.

Phenotypic assays are based on the measurement of virus growth inhibition in the presence of antiviral drugs. Various concentrations of virus are incubated with various concentrations of antiviral drugs, and the determination of the reduction of virus-induced cytopathic effect or plaque formation compared to a reference strain or the strain isolated before treatment enables the measurement of viral susceptibility to antiviral drugs. The gold standard phenotypic method for the evaluation of HSV susceptibility is the plaque reduction assay [60,62].

Although TK is not essential for growth in cell culture, it is important for viral pathogenesis, particularly for reactivation from latently infected trigeminal ganglia in animal models [68,69]. This feature has likely minimized the development of TK based resistance in the immunocompetent community. In patients with ACV resistant strain, cessation of antiviral treatment results in reversion of HSV isolates to ACV sensitivity [70]. The most frequent strains reactivated after an episode caused by a resistant HSV strain are thus ACV-sensitive [70]. However reactivation of some TK-negative HSV clinical isolates have been reported [71,72]. Therefore, despite an initial antiviral efficacy, the same resistance will likely be selected as the previous episode and ACV treatment may fail, especially if the immunosuppression condition remained.

## Conclusion

Laboratory confirmation of clinically suspected genital herpes diagnosis is necessary. In addition to helping the therapeutic management of ulcerative genital lesions and herpes diagnosis, it helps identify persons at risk of transmitting infection. Direct diagnosis is recommended and validated molecular assays are a good alternative to cell culture. Indirect diagnosis should use only FDA or CE approved type-specific serology based on glycoprotein G1 and G2 antigens and has to be considered for recurrent genital symptoms or atypical symptoms without laboratory confirmation and for testing pregnant women at risk of acquiring HSV infection.

## Competing interests

The authors declare that they have no competing interests.

## Authors’ contributions

JL and LB performed literature searches and drafted the manuscript. HP participated in editing the manuscript. All authors read and approved the final manuscript.
