# Erratum to: Diagnosis of genital herpes simplex virus infection in the clinical laboratory

**DOI:** 10.1186/s12985-015-0382-5

**Published:** 2015-10-13

**Authors:** Jérôme LeGoff, Hélène Péré, Laurent Bélec

**Affiliations:** Université Paris Diderot, Sorbonne Paris Cité, Microbiology laboratory, Inserm U941, Hôpital Saint-Louis, APHP, 1 Avenue Claude Vellefaux, Paris, 75010 France; Laboratoire de Microbiologie, hôpital Européen Georges Pompidou, Assistance Publique-Hôpitaux de Paris, Sorbonne Paris Cité, Paris, France; Faculté de Médecine Paris Descartes, Université Paris Descartes (Paris V), Sorbonne Paris Cité, Paris, France

## Erratum

We recently published a review on genital herpes diagnosis in the *Virology Journal* entitled “Diagnosis of genital herpes simplex virus infection in the clinical laboratory” [1]. Tables 1 and 2 were adapted from an article of Domeika and colleagues published in *Eurosurveillance* journal [2], with the formal permission of the Editor of *Eurosurveillance*. However, the article of Domeika and colleagues was not correctly cited as a source, due to a typographical error, and the correct reference is [18] (and not [9]) for both Table 1 and 2. Furthermore, it came to light that some sentences in the Abstract and the main body of the article were duplicated from previously published articles, including Strick et al., 2006 [3–8]. We apologize for any inconvenience caused.

The fundamental aim and basic purpose of our extensive and original review on genital herpes diagnosis was to give up-to-date information regarding the laboratory diagnosis of genital herpes that medicine laboratory needs to implement best assays in their diagnosis strategy [1]. We provide information about HSV-1 and HSV-2 characteristics that are critical for both serological and direct diagnosis (*Key structure elements for diagnosis*).

We provided information regarding the collection, transport and storage of clinical specimens for herpes diagnosis that have to be respected to ensure a good sensitivity. In the field of molecular assays, we summarized the state of the art including the most recent publications on Food and Drug Administration (FDA)-approved assays to help in the selection of a technique that is the most suitable according to the laboratory equipment and organization. We presented the current FDA approved serological assays with specific comments on the interpretation of index values and gave detailed guideline for the use of serological assays in clinical practice. Finally, for patients under antiviral treatment, we presented a practical guideline for therapeutic monitoring and summarized in a one table all the resistance mutations known for HSV-1 and HSV-2.
